# GMP Manufacturing and IND-Enabling Studies of a Recombinant Hyperimmune Globulin Targeting SARS-CoV-2

**DOI:** 10.3390/pathogens11070806

**Published:** 2022-07-19

**Authors:** Rena A. Mizrahi, Wendy Y. Lin, Ashley Gras, Ariel R. Niedecken, Ellen K. Wagner, Sheila M. Keating, Nikita Ikon, Vishal A. Manickam, Michael A. Asensio, Jackson Leong, Angelica V. Medina-Cucurella, Emily Benzie, Kyle P. Carter, Yao Chiang, Robert C. Edgar, Renee Leong, Yoong Wearn Lim, Jan Fredrik Simons, Matthew J. Spindler, Kacy Stadtmiller, Nicholas Wayham, Dirk Büscher, Jose Vicente Terencio, Clara Di Germanio, Steven M. Chamow, Charles Olson, Paula A. Pino, Jun-Gyu Park, Amberlee Hicks, Chengjin Ye, Andreu Garcia-Vilanova, Luis Martinez-Sobrido, Jordi B. Torrelles, David S. Johnson, Adam S. Adler

**Affiliations:** 1 GigaGen, Inc., South San Francisco, CA 94080, USA; rmizrahi@gigagen.com (R.A.M.); agras@gigagen.com (A.G.); aniedecken@gigagen.com (A.R.N.); ewagner@gigagen.com (E.K.W.); skeating@gigagen.com (S.M.K.); nikon@gigagen.com (N.I.); manickam.vishal.a@gmail.com (V.A.M.); masensio@gigagen.com (M.A.A.); jacksonleong@gmail.com (J.L.); amedinacucurella@gigagen.com (A.V.M.-C.); ebenzie@gigagen.com (E.B.); kcarter@gigagen.com (K.P.C.); ychiang@gigagen.com (Y.C.); robert@drive5.com (R.C.E.); rleong@gigagen.com (R.L.); ylim@gigagen.com (Y.W.L.); jsimons@gigagen.com (J.F.S.); mspindler@gigagen.com (M.J.S.); kstadtmiller@gigagen.com (K.S.); nwayham@gigagen.com (N.W.); seasquirtdoctor@gmail.com (D.S.J.); 2 Alira Health, Inc., Framingham, MA 01702, USA; wendy.lin@alirahealth.com (W.Y.L.); steve.chamow@alirahealth.com (S.M.C.); chuck.olson@kathario.com (C.O.); 3 Grifols S.A., 08174 Sant Cugat del Vallès, Spain; dirk.buscher@grifols.com (D.B.); jose.terencio@grifols.com (J.V.T.); 4 Vitalant Research Institute, San Francisco, CA 94118, USA; cdigermanio@vitalant.org; 5 Population Health Program, Texas Biomedical Research Institute, San Antonio, TX 78227, USA; ppinotamayo@txbiomed.org (P.A.P.); ahicks@txbiomed.org (A.H.); agarciavilanova@txbiomed.org (A.G.-V.); lmartinez@txbiomed.org (L.M.-S.); jtorrelles@txbiomed.org (J.B.T.); 6 Disease Intervention and Prevention Program, Texas Biomedical Research Institute, San Antonio, TX 78227, USA; jpark@txbiomed.org (J.-G.P.); cye@txbiomed.org (C.Y.)

**Keywords:** recombinant hyperimmune, GMP manufacturing, SARS-CoV-2

## Abstract

Conventionally, hyperimmune globulin drugs manufactured from pooled immunoglobulins from vaccinated or convalescent donors have been used in treating infections where no treatment is available. This is especially important where multi-epitope neutralization is required to prevent the development of immune-evading viral mutants that can emerge upon treatment with monoclonal antibodies. Using microfluidics, flow sorting, and a targeted integration cell line, a first-in-class recombinant hyperimmune globulin therapeutic against SARS-CoV-2 (GIGA-2050) was generated. Using processes similar to conventional monoclonal antibody manufacturing, GIGA-2050, comprising 12,500 antibodies, was scaled-up for clinical manufacturing and multiple development/tox lots were assessed for consistency. Antibody sequence diversity, cell growth, productivity, and product quality were assessed across different manufacturing sites and production scales. GIGA-2050 was purified and tested for good laboratory procedures (GLP) toxicology, pharmacokinetics, and in vivo efficacy against natural SARS-CoV-2 infection in mice. The GIGA-2050 master cell bank was highly stable, producing material at consistent yield and product quality up to >70 generations. Good manufacturing practices (GMP) and development batches of GIGA-2050 showed consistent product quality, impurity clearance, potency, and protection in an in vivo efficacy model. Nonhuman primate toxicology and pharmacokinetics studies suggest that GIGA-2050 is safe and has a half-life similar to other recombinant human IgG1 antibodies. These results supported a successful investigational new drug application for GIGA-2050. This study demonstrates that a new class of drugs, recombinant hyperimmune globulins, can be manufactured consistently at the clinical scale and presents a new approach to treating infectious diseases that targets multiple epitopes of a virus.

## 1. Introduction

Plasma hyperimmune globulin drugs have historically been used for emergency treatment during viral infection when other treatments are not available. For the severe acute respiratory syndrome associated coronavirus 2 (SARS-CoV-2), serum/plasma units were collected from individuals who recovered from coronavirus disease 2019 (COVID-19) and these drugs were used as the first line of treatment for infected individuals [1,2]. Hyperimmune globulins have significant advantages over monoclonal antibodies (mAbs); the use of purified plasma antibodies from convalescent donors or vaccinated animals has a demonstrated long history of successfully treating pathogens, including hepatitis B virus and rabies [3]. Mixtures of antibodies are beneficial for infectious diseases due to the diverse strains and epitopes that are targeted; however, plasma-derived hyperimmune globulins have many limitations. For example, the procurement of human plasma requires many donors with high anti-pathogen titers, which can be expensive and difficult to acquire in large quantities. Plasma is acquired from many donors at various titers, resulting in a plasma hyperimmune globulin titer that may be only two- to four-fold higher potency than plasma-derived intravenous immunoglobulin (IVIG) [4].

In recent studies, it has been determined that high potency plasma-derived or recombinant mAb drugs are effective in prophylactically blocking infection and therapeutically preventing progression to serious disease in high-risk COVID-19 populations [5,6]. Several mAbs have shown clinical efficacy for treating COVID-19 patients [7,8]. However, single mAb therapies have led to viral evasion around the neutralizing epitopes, resulting in a reduction or abrogation of efficacy [9,10,11]. Thus, variable viral targets require not only potent but also multivalent antibody therapeutics to efficiently block infection and prevent viral evolution. It is impossible to predict future variants of SARS-CoV-2, so drugs that are robust over the long term should have extremely broad epitope coverage.

**Figure 1:**
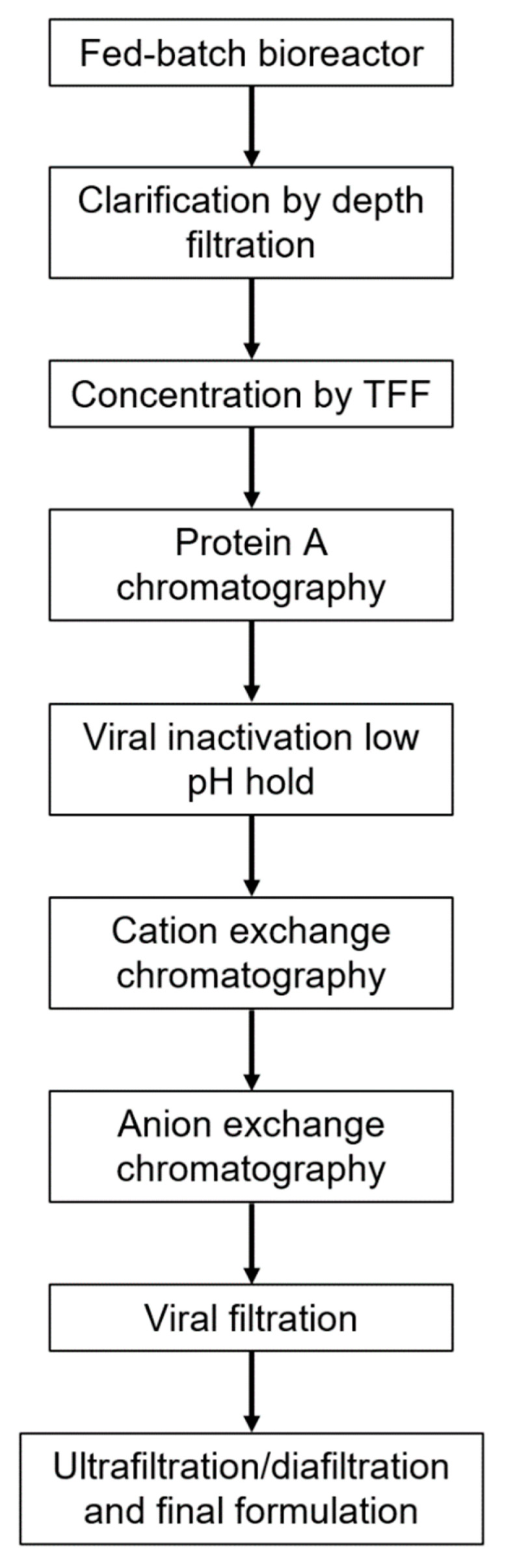
Process flow diagram outlining the upstream and downstream manufacturing process for GIGA-2050, which is similar to a standard mAb process. After fed-batch production in a conventional single-use bioreactor, the process includes clarification via depth filtration, concentration of the clarified harvest followed by Protein A chromatography, low pH viral inactivation, cation exchange chromatography in bind and elute mode, anion exchange chromatography in flow through mode, viral filtration and ultrafiltration/diafiltration to concentrate, and buffer exchange into the final formulation.

Previously, we generated the world’s first highly diverse, recombinant hyperimmune globulins; using a high-throughput single B cell capture microfluidic technology, diverse antibody repertoires from COVID-19 convalescent donors were captured and antibody sequences were cloned and expressed in a targeted integration Chinese hamster ovary (CHO) cell line [12]. Upstream and downstream processes were developed based on standard mAb manufacturing, to allow for use of existing manufacturing infrastructure (Figure 1). The recombinant hyperimmune globulin drug product, GIGA-2050, is currently in clinical trials in hospitalized COVID-19 patients in the United States (US). We previously demonstrated that GIGA-2050 comprises 12,500 unique antibodies and binds to a wide variety of SARS-CoV-2 variants [12], like plasma hyperimmune globulins for COVID-19; a detailed study of the ability of GIGA-2050 to neutralize all major variants of concern is underway (manuscript in preparation).

In this current study, we describe the manufacturing controls, pharmacological assessments, and in vivo studies used to support an investigational new drug (IND) application. To assess consistency across batches and upstream process scalability, the CHO cells were monitored for cell viability, antibody production titers, and sequence diversity. Additionally, each GIGA-2050 lot was tested for purity and product quality, including potency using a SARS-CoV-2 Spike ELISA and a cell-based pseudovirus neutralization assay. This work demonstrates that a recombinant hyperimmune globulin can be manufactured under GMP at up to 250 L scale, leveraging equipment and methods used in conventional mAb manufacturing, and that such a process was comparable across multiple lots. Nonhuman primate (NHP) toxicology and pharmacokinetics (PK) studies, also called toxicokinetics (TK), were performed to investigate safety and PK of this drug in preparation for IND submission to the US Food and Drug Administration (FDA) for first-in-human (FIH) clinical studies.

## 2. Results

**Figure 2:**
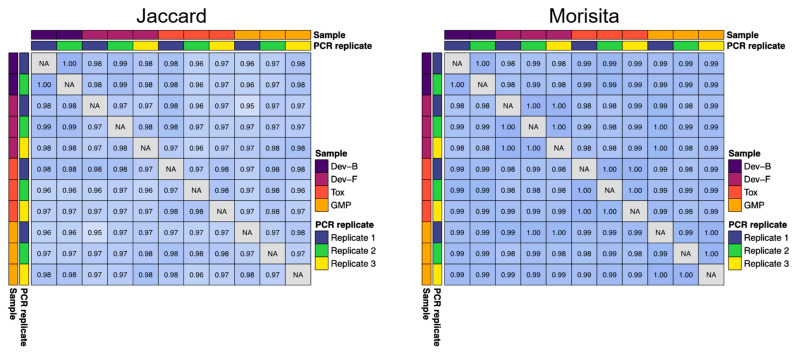
Jaccard and Morisita statistical analyses of antibody RNA-Seq data show a high degree of sequence similarity (>0.95 for Jaccard and >0.98 for Morisita) between two representative development (Dev) lots, the Tox lot, and the GMP lot. Analysis of PCR replicates from these lots found that the lot-to-lot variability was no greater than the variability across PCR replicates from a single lot, indicating strong batch-to-batch consistency.

Several GIGA-2050 fed-batch production lots were generated from the research cell bank (RCB) or master cell bank (MCB) (Supplementary Material Table S1). To quantify the upstream consistency of antibody sequence diversity across several of these batches, we computed Jaccard and Morisita indices using antibody repertoire (RNA-Seq) data. The Morisita index considers the abundance of a given sequence in the sample, whereas the Jaccard index does not. We directly compared the 200 L toxicology (Tox), 200 L development (Dev-F), and 250 L GMP (GMP) lots to a representative development lot (Dev-B) (Figure 2). When looking at all antibodies present at an abundance of 0.01% or higher, the overlap between all pairs of samples according to the Jaccard index was >0.95, and the overlap between all pairs of samples according to the Morisita index was >0.98. We analyzed PCR replicates from these lots (either duplicate or triplicate PCR reactions amplified from a single RNA sample from each lot) and found that the lot-to-lot variability was no greater than the variability across PCR replicates from a single lot, indicating strong qualitative and quantitative consistency in antibody content between development, toxicology, and GMP lots (Figure 2).

Stability of the mock MCB was evaluated over 20 passages (>70 generations; Figures S1–S4). At approximately every six passages (P#), a satellite fed-batch study was conducted. Genetic stability (copy number), cell growth (viable cell density, VCD), productivity (titer), and product quality of Protein A purified material (SEC-HPLC, CE-SDS, and anti-SARS-CoV-2 S1 binding ELISA) were assessed. The P0, P6, P12, and P20 stability cell banks were comparable for cell growth, viability, and titer. The P0 stability cell bank was within 4% of the P20 stability cell bank peak VCD on day 9 and 6% of the P20 stability cell bank final titer (Table S2). The purity by SEC-HPLC changed by no more than 1% between P0 and P20, although the purity as measured by CE-SDS did decrease 6.3% (reduced) and 11.8% (non-reduced) between the same samples. Most of the impurities detected would be removed in the polishing steps of the downstream process. There was also a decrease in potency of 25.5% from P0 to P20, but between P0 and P12 there was a 12.8% increase in potency. When the potency assay was validated for GMP lot release, the accuracy was assessed through repeated tests of the same product in the range of 70–130% relative potency; within this relative potency range, the percent bias ranged from −6% to 24%. Additionally, the acceptance criterion for precision of the relative potency measurements during the validation study was a coefficient of variation of ≤30%, which was achieved. Since up to 30% variation is expected in this method, the potency variation seen with these stability samples is likely due to assay variability. Overall, the cell culture process performance assessment demonstrates with a high level of confidence that the GIGA-2050 cell line is stable up to approximately 79 generations, which provides appropriate coverage for Phase 1 GMP 250 L fed-batch production.

To monitor downstream process consistency, we performed a series of analyses on downstream in-process intermediates from the Dev-F (200 L) and GMP (250 L) lots. The focus of these analyses was on clearance of product- and process-related impurities, to assess the comparability of the downstream processes performed at different facilities. The assays included concentration by UV A280 or Protein A-HPLC, SEC-HPLC, CE-SDS (non-reduced and reduced), CHO host cell protein ELISA, and CHO host cell DNA qPCR. The intermediates assessed included the concentrated clarified harvest (CCH), depth filtered neutralized viral inactivated Protein A pool (VI pool), and cation exchange eluate (CEX eluate). Results of all analyses were comparable between the two lots (Tables S3–S5), proving that the Dev-F and GMP lots represent comparable processes.

To assess the consistency of the final protein product quality, standard lot release assays for recombinant mAbs, plasma hyperimmune globulins, and anti-SARS-CoV-2 drugs were used. A subset of the key data for all lots is shown in Table 1, including purity and potency assays. Both process-related (CHO host cell protein and CHO host cell DNA) and product-related (aggregate and low molecular weight species) impurities were present in low amounts in all batches. The 95% confidence intervals for SARS-CoV-2 binding ELISA fell between 0.028 and 0.054 µg/mL across the eight lots. Reference NIBSC plasma, a reference mAb, and a SARS-CoV-2 plasma hyperimmune globulin had 95% confidence intervals of 8.79–9.78 µg/mL, 0.097–0.11 µg/mL, and 27.1–29.2 µg/mL, respectively, suggesting that the binding strength of GIGA-2050 is similar to a mAb but much stronger than existing plasma-derived drug products. The 95% confidence intervals for the pseudotype neutralization potency assay fell between 0.28 and 0.53 µg/mL across the eight lots. Reference NIBSC plasma, a reference mAb, and a SARS-CoV-2 plasma hyperimmune globulin had 95% confidence intervals of 10.6–20.2 µg/mL, 0.46–0.52 µg/mL, and 220.1–273.9 µg/mL, respectively. Thus, GIGA-2050 has neutralizing activity which is closer to a mAb than plasma-derived drug products. Prior work analyzing neutralizing titer of 16 lots of anti-SARS-CoV-2 plasma hyperimmune globulin (Takeda) suggests that plasma drug lots vary in neutralizing potency by as much as five-fold [13]. Thus, although further process development may further improve the consistency in binding and potency between lots of GIGA-2050, the manufacturing process for GIGA-2050 already produces higher lot consistency than certain plasma hyperimmune manufacturing processes.

Conventional in vitro assessments did not detect significant off-target effects for GIGA-2050. Anti-HLA for the GIGA-2050 GMP lot was measured and resulting in CL-1 = 45.6 and CL-II = 12.5, which is below the assay reactivity cutoff values (Class-I = 59.3; Class-II = 27.5). Compendial hemagglutination assays for anti-A, anti-B, and anti-D were all negative for the GMP lot.

In a human tissue microarray study of 37 tissues (Table S6), the Tox GIGA-2050 lot stained the cytoplasm of the mononuclear cells from three individual’s placenta and one individual’s cerebellum at both 2.5 µg/mL and 25 µg/mL, and one individual’s bladder, pituitary, liver (Kupffer cells), pancreas, and spleen at 25 µg/mL only; the staining intensity and frequency were “1+” and “rare” to “rare to occasional” at 2.5 µg/mL, and “1+” and “rare” to “occasional” at 25 μg/mL. There was no GIGA-2050 staining observed in the other human tissues. IVIG stained the cytoplasm of the mononuclear cells from three individual’s placenta and one individual’s cerebellum at both 2.5 µg/mL and 25 µg/mL, and one individual’s bladder, pituitary, liver (Kupffer cells), pancreas, and spleen at 25 µg/mL only; the staining intensity and frequency were “1+” and “rare” to “rare to occasional” at 2.5 µg/mL, and “1+” to “2+” and “rare” to “occasional to frequent” at 25 µg/mL. There was no IVIG staining observed in the other human tissues. Taken together, these results indicate that GIGA-2050 comprises no more significant non-specific human tissue binding than IVIG.

The primary purpose of the NHP TK study was to determine the potential toxicity of GIGA-2050 when administered by IV infusion to cynomolgus monkeys for a single dose (25, 125, or 475 mg/kg), and to identify a no observable adverse effect level (NOAEL) that demonstrated an acceptable safety margin to support the proposed human doses. There were no unscheduled deaths or adverse events noted during the TK study. Test article-related changes in clinical observation, local irritation, body weight, food consumption, body temperature, ophthalmic examinations, clinical pathology (hematology, coagulation, coagulation, and urinalysis), safety pharmacology (electrocardiography, blood pressure, heart rate, respiration, and neurological examinations), cytokine analysis, or pathology changes (organ weights and macroscopic and microscopic observations) were not noted between the vehicle negative control group and the GIGA-2050 dose groups. Results of this study showed that a single dose of GIGA-2050 administered IV was well tolerated in the cynomolgus macaque at doses up to 475 mg/kg, and thus the NOAEL for this study was 475 mg/kg/dose.

The secondary goal of the NHP TK study was to assess pharmacology of GIGA-2050 through PK measurements using blood draws throughout the study. There were no observed PK differences between male and female animals (Table S7, Figure S5). In general, PK parameters were as expected compared with human IgG1 infused in cynomolgus observed elsewhere; for example, across the treatment groups, the median T1/2 was 300 h, which is within the range of 264–502 h observed elsewhere for human IgG1 mAbs [14].

**Figure 3:**
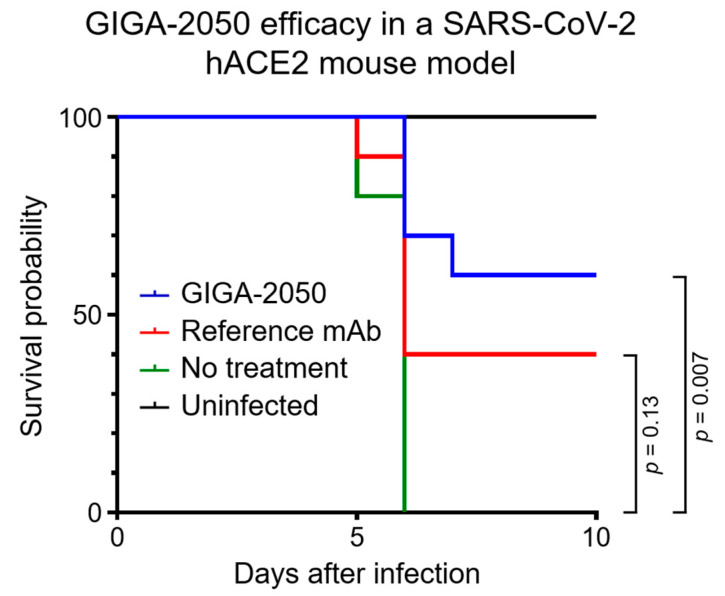
Kaplan–Meier curves for K18 hACE2 transgenic mice after SARS-CoV-2 infection. The survival probability is reported for uninfected controls (black), infected but no treatment control (green), reference mAb (CC12.3; 1.5 mg/kg; red), and GIGA-2050 (5 mg/kg; blue). Day after challenge with SARS-CoV-2 is on the x-axis. Animals were treated with GIGA-2050 or the reference mAb 24 h before infection with SARS-CoV-2. For treatment with GIGA-2050, the probability of survival is significantly higher than the no treatment infected control (*p* = 0.007, Mantel–Cox). The reference mAb at the concentration studied (1.5 mg/kg) did not significantly reduce mortality compared to no treatment control (*p* = 0.13, Mantel–Cox).

In an in vivo efficacy study with a K18 hACE2 transgenic mouse model that was highly pathogenic after infection with SARS-CoV-2 [15], GIGA-2050 was found to significantly reduce mortality compared to the no treatment control group (Figure 3, *p* = 0.007). By Day 6 after inoculation with SARS-CoV-2, all mice in the no treatment/infected control group were deceased. In contrast, 6/10 mice survived in the GIGA-2050 treatment group at the end of the study (Day 10 after infection). The control anti-SARS-CoV-2 mAb did not significantly decrease mortality (Figure 3, *p* = 0.13), presenting a 40% survival. Weight loss (morbidity) for all animals was highest at 6 days after infection; surviving animals gained weight to the same range as the normal uninfected control group by 10 days after infection (Figure S6). To investigate whether there were differences in weight change among groups in the first few days after infection, weight change was calculated by subtracting the weight on Day 3 from the weight on Day 0, and then these changes were compared between the treatment arms using the nonparametric Wilcoxon rank sum test. No statistically significant differences were observed in this analysis.

## 3. Discussion

GIGA-2050 is the first example of a new class of drugs, recombinant polyclonal hyperimmune globulins. Prior to this work, pioneering groups have worked on manufacturing methods for much smaller mixtures of recombinant antibodies [16]. Unlike GIGA-2050, these prior methods involved creation of separate MCBs for each mAb, followed by mixing the MCBs prior to bioproduction. For example, rozrolimupab was a mixture of 25 antibodies, whereas GIGA-2050 consists of >10,000 different antibodies. Rozrolimupab completed a Phase 2 study for treatment of primary immune thrombocytopenia [17] but has apparently been discontinued by the manufacturer. Our methods uniquely enable large-scale GMP production of mixtures of thousands of antibodies.

As multivalent antibody therapeutics, recombinant hyperimmune globulins represent a potent treatment for infectious disease that decreases the likelihood of antigenic escape. Although this is a new class of drug, it will not require construction of new manufacturing facilities; instead, we have shown that GIGA-2050 can be manufactured using existing infrastructure already in place for manufacturing of mAbs. In 2011, the global capacity for mammalian cell production in bioreactors was approximately 0.5 million liters [18]. Capacity has continued to grow over the past decade, so should the need arise, manufacturing of a recombinant hyperimmune globulin could be scaled to produce millions of doses.

We have demonstrated that a conventional mAb platform approach can be applied to a recombinant hyperimmune globulin product to achieve desired separation of aggregates and process related impurities. The platform production process has produced the GIGA-2050 drug substance and drug product lots at different scales and manufacturing sites that have demonstrated no substantial differences in the purity measurements and impurity clearance for in-process steps and analytically comparable characteristics and quality attributes of the final drug substance. In the future, we hope to apply similar methods to larger scale GMP runs, for example, bioreactors with a capacity of 1000 L or higher.

GIGA-2050 nonclinical studies, which were supportive of an IND application to the FDA, included in vitro evaluation of GIGA-2050 pharmacology via anti-SARS-CoV-2 binding and neutralization. The GLP TK evaluation of GIGA-2050 was conducted in a single-ascending dose study in cynomolgus macaques. Results of the nonclinical studies demonstrated that GIGA-2050 is expected to be a potent neutralizer of SARS-CoV-2 at a dose level of 5 mg/kg in humans and was well-tolerated with no observed adverse effects up to, and including, a dose level of 475 mg/kg IV, the NOAEL determined in the cynomolgus macaque TK study. Taken together, results supported the starting dose of 5 mg/kg for a Phase 1 FIH study in patients with COVID-19 caused by SARS-CoV-2 infections. In vivo studies in mice using the 5 mg/kg concentration of GIGA-2050 further proves that this compound protects (60% survival) against a lethal SARS-CoV-2 infection. 

GIGA-2050 for treating pandemic SARS-CoV-2 infection is the first recombinant hyperimmune globulin made at the industrial scale. Altogether these results present the precedent for manufacturing recombinant polyclonal hyperimmune globulin drugs for rapid deployment of passive immune therapeutics for protection of populations at risk and treatment after exposure during pandemic and endemic infectious disease transmission.

In conclusion, our work demonstrates that recombinant hyperimmune globulins can be manufactured consistently at the clinical scale. This new class of drugs presents a new approach to treating infectious diseases, targeting multiple epitopes of pathogens to decrease the likelihood of new variants escaping the drug targets.

## 4. Materials and Methods

### 4.1. Master Cell Bank Generation and Production

The production of the GIGA-2050 research cell bank (RCB) was described previously [12]. In brief, single B cells from COVID-19 convalescent donors were captured in droplets using a microfluidic device and combined with a mixture of lysis buffer and oligo dT beads (NEB, Ipswich, MA, USA). The mRNA-bound beads were purified before an emulsion was created using OE-RT-PCR reagents and the beads as the template. The IgK and IgG variable regions were amplified by PCR and linked together to form a single chain variable fragment (scFv) so that the product could be used for deep sequencing, yeast display libraries for antigen-enrichment, or full-length CHO expression. Separately, the suspension CHO line CHOZN^®^ GS−/− (MilliporeSigma, St. Louis, MO, USA) was used to create a targeted integration host cell line for recombinant hyperimmune production. The scFv was cloned into a vector backbone and the constant regions were added and cloned through two Gibson Assemblies into expression plasmids. The resulting plasmid library was transfected into the targeted integration cell line to enable production of antibody heavy and light chains. The targeted integration cell line was used so that each transfected CHO cell produces only one antibody, preventing the Ig chain mispairing that would arise if multiple antibodies were expressed by a single CHO cell.

To produce the GMP master cell bank (MCB) from the RCB, a single vial of RCB at a viable cell density (VCD) of 2.0 × 10^7^ cells per mL was thawed and seeded into EX-CELL CD CHO Fusion growth medium (Sigma-Aldrich, St. Louis, MO, USA). Cells were cultured in flasks at 37.0 ± 1 °C under 5 ± 1% CO_2_ and 80% humidity shaking incubator. Expansion took place over a period of 9 days with cells sub-cultured at 3–5 × 10^5^ viable cells/mL every 3 days when VCD reached 4–5 × 10^6^ cells per mL. Cryogenic vials were filled with 1.0 mL of Cryostor CS-10 (Stemcell Technologies, Vancouver, BC, Canada) at a concentration of 2.0 × 10^7^ viable cells per mL.

The RCB was used to produce GIGA-2050 lots Dev-A, Dev-B, Dev-C, and Dev-D. The MCB was used to produce GIGA-2050 lots Dev-E, Dev-F, Tox, and GMP (Table S1).

### 4.2. Master Cell Bank Stability

The stability of GIGA-2050 was evaluated by comparing the cell growth and productivity at different cell ages. Cell ages were calculated as population doubling level (PDL) using the following equation and passaged for approximately 79 generations.

$$\mathrm{PDL}=\frac{\log\left({\mathrm{VCD}}_{\mathrm{final}}\right)-\;\log\left({\mathrm{VCD}}_{\mathrm{initial}}\right)}{\log\left(2\right)}$$

VCD = viable cell density (viable cells/mL).

A mock MCB (mMCB) was generated at GigaGen from the RCB to mimic the cell age of the MCB generated at Eurofins (Lancaster, PA, USA). The mMCB was used as passage 0 (P0, PDL 0). From P0, a stability cell bank was created after 20 days at passage 6 (P6, PDL 23), another stability bank was created after an additional 21 days at passage 12 (P12, PDL 47), and a final stability cell bank after another 28 days at passage 20 (P20, PDL 79). The mock MCB P0, P6, P12, and P20 stability cell banks were thawed and expanded for a 15-day seed train in parallel and evaluated in a stability shake flask fed-batch production process in duplicate (Figure S1).

The stability shake flask fed-batch production process evaluation was performed at 250 mL shake flask scale. All stability fed-batch evaluations were inoculated at 0.4 × 10^6^ VCD and a starting volume of 50 mL using EX-CELL Advanced Fed-Batch Medium (MilliporeSigma, St. Louis, MO, USA) as production media. The fed-batch cultures were grown in a shaking incubator (Kuhner, Basel, Switzerland) with parameters set at 37 °C, 5% CO_2_, 80% humidity, and 125 RPM (25 mm orbital throw). On Day 4, when cultures reached at least 10 × 10^6^ VCD, the temperature was dropped to 32 °C for the remainder of the production. Cultures were supplemented, calculated from starting volume, with 2% Cellvento 4Feed COMP (MilliporeSigma, St. Louis, MO, USA) and 4% EX-CELL Advanced CHO Feed 1 (MilliporeSigma, St. Louis, MO, USA) as bolus nutrients additions on Days 3, 5, 7, 9, and 11. A 45% glucose solution (MilliporeSigma, St. Louis, MO, USA) was added up to 6 g/L when offline glucose measurements were ≤4 g/L. Throughout the 14-day process, cell suspension samples were analyzed for cell growth and viability by the Countess II (Thermo Fisher Scientific, Waltham, MA, USA) and titer using the Cedex BioAnalyzer (Roche, Basel, Switzerland). When the production culture reached Day 14 with viability > 70%, the culture was centrifuged and sterile filtered through a 0.22 µm PES membrane. Replicate PDL samples were combined and frozen at −80 °C for further downstream processing.

In addition to analyzing growth, viability, and titer, the stability study also analyzed transgene copy numbers. Cells for each PDL were taken and gDNA was extracted for analysis in the CNV assay. However, the sequence diversity was not determined for these samples, which is something that would be of interest to investigate in future studies.

The material harvested from the stability study was purified over Protein A affinity chromatography and formulated in the same buffer as the final product. Protein A affinity chromatography does not provide significant removal of aggregates, thus any changes in product quality due to cell line aging was observable at this stage. The purification of the final material included several additional polishing steps that remove most of the impurities observed. The purified material was assayed by SEC-HPLC, CE-SDS, and anti-SARS-CoV-2 S1 binding ELISA (Figures S2–S4).

### 4.3. Manufacturing GIGA-2050

When developing the GIGA-2050 manufacturing process, the aim was to model it on a typical mAb manufacturing platform approach, to take advantage of existing expertise and infrastructure in the contract manufacturing and development space. The strategy to manufacture GIGA-2050 can be used to manufacture any recombinant hyperimmune globulin at any site that manufactures mAbs, as outlined in Figure 1. The GIGA-2050 upstream process specifies a 15-day seed train for any scale production to control for cell age and a 14-day fed-batch process in a standard single-use bioreactor, with supplementation of feeds and glucose, to achieve consistent diversity and potency of the recombinant hyperimmune globulin.

The downstream recovery process employs a series of standard mAb chromatography purification steps and viral reduction steps. Protein A capture chromatography in bind and elute mode is followed by low pH viral inactivation. Several polishing steps are used including cation exchange chromatography (CEX) in bind and elute mode for reducing residual host cell proteins (HCPs) and other product-related impurities and anion exchange (AEX) in flow-through mode to further remove residual HCPs, residual host-cell DNA, and putative viruses. A viral filtration step and ultrafiltration and diafiltration (UF/DF) are performed prior to final formulation.

#### 4.3.1. Upstream

A consistent seed train protocol was performed for all scales comprising a sufficient number of cell doublings to allow for inoculation of a bioreactor at up to 2000 L scale. For each bioreactor production run, a single vial of GIGA-2050 cell bank (either RCB or MCB) was thawed into EX-CELL CD CHO Fusion media (MilliporeSigma, St. Louis, MO, USA). The entire volume of cells was seeded into a 250 mL non-baffled, vented shake flask at a final volume of 50 mL. The shake flask was incubated at 37 °C, 5% CO_2_, 80% humidity, and 125 RPM (25 mm orbital diameter) or 145 RPM (19 mm orbital diameter). Three days post thaw, the VCD was 4.0–6.0 × 10^6^ vc/mL with a viability ≥ 90%. At this point, the cells were passaged using EX-CELL CD CHO Fusion at a seeding density of 0.4 × 10^6^ vc/mL. In a similar manner, the culture was expanded three more times. The final passage before fed-batch inoculation was done in EX-Cell Advanced CHO Fed Batch media (MilliporeSigma, St. Louis, MO, USA).

Three days after completing the fifth passage, fed-batch production vessels were seeded (see Table S1 for vessel type used for each GIGA-2050 lot). Each bioreactor or shake flask was seeded at a VCD of 0.4 ± 0.1 × 10^6^ vc/mL in EX-Cell Advanced CHO Fed Batch media and was controlled using the following set points: temperature setpoint Days 0–4 setpoint 37 °C; temperature Days 4–14 setpoint 32 °C; dissolved oxygen setpoint 30%; pH Days 0–3 7.0 ± 0.2; pH Days 3–14 7.0 ± 0.1. For lot Dev-F, the pH for Days 0–3 was controlled at 7.05 ± 0.15, while the same setting was used for Days 3–14 as the other bioreactors. For lot Tox, the temperature was shifted to 32 °C on Day 6. EX-Cell Advanced CHO Feed (MilliporeSigma, St. Louis, MO, USA) and Cellvento 4Feed COMP (MilliporeSigma, St. Louis, MO, USA) were added on Days 3, 5, 7, 9, and 11. Feed volumes were determined as a percentage of the current bioreactor volume, such that EX-Cell Advanced CHO Feed 1 was added at 4% of the volume of the bioreactor and Cellvento 4Feed COMP was added at 2% of the volume of the bioreactor. For lot Tox, the feed on Day 5 was reduced to 2% EX-Cell Advanced CHO Feed 1 and 1% Cellvento 4Feed COMP. The fed-batch production for this lot included an additional feed of 4% EX-Cell Advanced CHO Feed 1 and 2% Cellvento 4Feed COMP on Day 15. Glucose levels were monitored daily, and starting on Day 3 were maintained above 4 g/L by adding a 45% Glucose solution until levels reached 6 g/L. The bioreactors were harvested after 14 days of culture using either depth filtration or centrifugation, except for lot Tox which was extended to 16 days. Around 5 to 10 million cells were collected from each bioreactor on the day of harvest for antibody repertoire sequencing.

#### 4.3.2. Downstream

Prior to purification, the harvested cell culture fluid (HCCF) for all lots except Dev-A was concentrated to 1.0–2.1 g/L using a 30 kDa molecular mass cutoff (MMCO) cellulose acetate tangential flow filtration cartridge (Repligen, Waltham, MA, USA). The first purification step used for GIGA-2050 was Protein A chromatography using MabSelect Sure PrismA resin (Cytiva, Marlborough, MA, USA). The column was equilibrated with 20 mM phosphate, 150 mM NaCl, pH 7.4, then loaded with harvested cell-culture fluid at 20–40 g/L, washed with 20 mM phosphate, 500 mM NaCl, pH 7.4 and 50 mM phosphate, pH 6.0, and eluted with 50 mM sodium acetate, pH 3.5. The pH of the Protein A eluate pool was adjusted to 3.5 using 1 M acetic acid and the material was held at this pH for 1-h to inactivate the putative virus, after which it was adjusted to pH 5 using 1 M Tris-HCl, pH 9 and filtered to remove particulates. This was followed by a CEX chromatography step using POROS XS resin (Thermo Fisher Scientific, Waltham, MA, USA). The column was equilibrated with 50 mM sodium acetate, pH 5.0, then loaded with the filtered neutralized low pH hold pool at 14–24 g/L, followed by washing with 50 mM sodium acetate, 100 mM sodium chloride, pH 5.0. The material was eluted over a 20-column volume gradient to 50 mM sodium acetate, 400 mM sodium chloride, pH 5.0. The eluate consisted of several distinct peaks, of which only the first was collected. The CEX eluate was diluted with 20 mM tris-acetate, pH 7.4 to <8 mS/cm, then run through a Sartobind Q AEX membrane (Sartorius, Göttingen, Germany) in flow through mode. For lots Dev-A, Dev-B, Dev-D, and Dev-E, the AEX flowthrough was concentrated using a 30 K MMCO cellulose acetate tangential flow filtration cartridge (MilliporeSigma, St. Louis, MO, USA), then diafiltered with 200 mM glycine pH 4.5 and sterile-filtered. For lots Dev-C, Dev-F, Tox, and GMP, the AEX flowthrough was flowed through a Planova 75 N pre-filter followed by a Planova BioEX viral filter. For these four lots, the viral filtrate was concentrated using a 30 K MMCO cellulose acetate tangential flow filtration cartridge (MilliporeSigma, St. Louis, MO, USA or Repligen, Waltham, MA, USA), then diafiltered with 200 mM glycine pH 4.5 and sterile filtered.

### 4.4. Deep Antibody Repertoire Sequencing by RNA-Seq

Deep antibody sequencing libraries were prepared and analyzed as described previously [12,19]. Libraries were sequenced on a MiSeq (Illumina, San Diego, CA, USA) using a 500 cycle MiSeq Reagent Kit v2, according to the manufacturer’s instructions. RNA was harvested from 5 to 10 million CHO cells that were harvested at the conclusion of the production runs. Tailed-end RT-PCR was used to add Illumina sequencing adapters to the 5′ and 3′ ends of the IgG heavy chain transcript. A median of 140,738 sequence reads were obtained for each sequencing library (range: 104,305 to 960,104). Sequence analysis, including error correction, reading frame identification, and FR/CDR junction calls, was performed as previously described [19]. Clones were defined as sequences with a unique CDR3H amino acid sequence. Jaccard and Morisita indices were calculated using the R package tcR (version 2.3.2) [20], using clones that represented ≥0.01% of a given sequencing library. Sequencing data are available in the Short Read Archive (SRA) under project identifier PRJNA784610.

### 4.5. Protein Product Characterization

#### 4.5.1. Analytical Biochemistry

Monomeric purity of GIGA-2050 was determined by size-exclusion chromatography (SEC-HPLC). GIGA-2050 was diluted in mobile phase (25 mM sodium phosphate buffer, 200 mM sodium chloride, pH 7.0) and injected into an HPLC-UV/diode array detector (DAD) system equipped with a size-exclusion column with dimensions of 7.8 × 300 mm, particle size of 2.7 µm, and a pore size of 300 Å (Agilent AdvanceBio, Santa Clara, CA, USA or equivalent). Injected GIGA-2050 was then eluted with 90% mobile phase and 10% acetonitrile (*v*/*v*). Following separation, the relative percentages of monomer, high, and low molecular weight species were quantified via UV detection.

Capillary electrophoresis sodium-dodecyl sulfate (CE-SDS) was performed under reducing and non-reducing conditions to assess product purity. Samples were run on a capillary electrophoresis system (Beckman Sciex PA-800 Plus, Brea, CA, USA or Agilent BioAnalyzer, Santa Clara, CA, USA), along with a protein molecular mass standard. The resulting electropherograms depict absorbance or fluorescence as a function of migration time. Purity of reduced samples was reported as percentage area of heavy chain plus light chain peaks as compared to the areas of all the peaks on the electropherogram. Purity of non-reduced samples was reported as the percentage area of the main peak as compared to the areas of all the peaks on the electropherogram.

#### 4.5.2. Residual Host Cell Impurities

Residual CHO HCP was measured by sandwich ELISA using a commercial CHO Host Cell Protein detection kit following the manufacturer’s instructions (F550-1; Cygnus Technologies, Southport, NC, USA). The standard curve was prepared by eight-step, two-fold dilution of the highest concentration standard in Cygnus sample diluent. Test articles were diluted into Cygnus sample diluent at least four-fold to satisfy the minimum required dilution. Mean responses of replicate standard curve levels were plotted against the log of the corresponding concentrations. A four-parameter nonlinear regression was applied to determine the best fit for the standard curve. Sample and control CHO-HCP levels were determined by comparison of their average response to the standard curve.

Host cell DNA samples were extracted using either the resDNASEQ quantitative CHO DNA kit (Applied Biosystems, Waltham, MA, USA) or the DNeasy Blood and Tissue kit (Qiagen, Hilden, Germany), along with positive control CHO DNA spike-in samples. Extracted samples were analyzed by qPCR using 2X Taqman Universal PCR Master Mix II (Applied Biosystems, Waltham, MA, USA) with primers and Taqman probes targeting CHO DNA. The results were reported in pg CHO DNA per mg of protein product.

#### 4.5.3. Anti-SARS-CoV-2 Binding ELISA

Anti-SARS-CoV-2 antibody reactivities were measured using a protocol based on published ELISA methods [21]. In brief, ELISA plates were coated at 2 μg/mL with SARS-CoV-2 Spike (Sino Biological, Wayne, PA, USA). Recombinant products, positive control mAb (CR3022; Absolute Antibody, San Diego, CA, USA), plasma-derived polyclonal anti-SARS-CoV-2 research reagent (20/130; NIBSC, Potters Bar, Hertfordshire, UK), anti-SARS-CoV-2 plasma hyperimmune globulin (Grifols, S.A., Sant Cugat del Vallès, Spain), and negative control IVIG (Gamunex; Grifols, S.A., Sant Cugat del Vallès, Spain) were serially diluted in sample buffer (1 × PBS + 0.05% Tween + 0.3% dry milk). After one-hour incubation, bound antibodies were detected using horseradish peroxidase (HRP)-conjugated mouse anti-human IgG (Jackson Immunoresearch, West Grove, PA, USA). Quantitative measurements were performed on a plate reader (Molecular Devices, Fremont, CA, USA) and analyzed using Prism (GraphPad, San Diego, CA, USA) to calculate the 50% effective concentration (EC50) of samples. The concentration of total IgG was calculated by UV280 measurement using an extinction coefficient of 1.5 (mg/mL)^−1^ cm^−1^. Triplicate measurements were made for each dilution. EC50 95% confidence interval calculations were performed using GraphPad Prism and the asymmetric profile-likelihood method.

#### 4.5.4. Anti-SARS-CoV-2 Neutralization Potency Assay

The SARS-CoV-2 neutralization potency assay was performed in 96-well plates using pseudotyped reporter viral particles (RVPs) and hACE2 expressing HEK-293T target cells (293T-hsACE2; Integral Molecular, Philadelphia, PA, USA) transiently transfected with TMPRSS2 expression plasmid. The green fluorescent protein (GFP) RVPs expressing Wuhan Hu-1 SARS-CoV-2 Spike (Integral Molecular, Philadelphia, PA, USA) were mixed with 2.5-fold serial dilutions of recombinant products, positive control mAb (SAD-S35; Acro Biosystems, Newark, DE, USA), plasma-derived polyclonal anti-SARS-CoV-2 research reagent (20/130; NIBSC, Potters Bar, Hertfordshire, UK), positive control, anti-SARS-CoV-2 plasma hyperimmune globulin (Grifols, S.A., Sant Cugat del Vallès, Spain), or negative control IVIG (Gamunex; Grifols, S.A., Sant Cugat del Vallès, Spain). After one-hour incubation, 4 × 10^4^ target cells were added to each well and incubated at 37 °C for 72 h. After incubation, the media was removed from all wells without disturbing the adherent cells. Cells were lifted by incubation with TrypLE (Thermo Fisher Scientific, Waltham, MA, USA) for 3 min at 37 °C. Following trypsinization, cells were washed, stained with 4′,6-diamidino-2-phenylindole (DAPI), and passed through a 30–40 μm filter (Pall Corporation, Port Washington, NY, USA) before quantifying GFP+ cells using a Cytoflex LX (Beckman Coulter, Indianapolis, IN, USA). Flow cytometry data were analyzed by FlowJo (BD Biosciences, San Jose, CA, USA). Triplicate measurements were made for each dilution. The 50% inhibitory concentration (IC50) 95% confidence interval calculations were performed using Prism (GraphPad, San Diego, CA, USA) and the asymmetric profile-likelihood method.

#### 4.5.5. Anti-HLA Assays

Screening tests for anti-HLA class I and II were performed by Vitalant Research Institute (San Francisco, CA, USA) with One Lambda LabScreen LSM12 (LabScreen Mixed; OneLambda, Los Angeles, CA, USA) multiantigen bead kits according to the manufacturer’s instructions. The assay measures the binding of the antibody to fluorescein tagged microbeads (six beads coated with purified class I antigens with up to 48 antigens per bead and three beads coated with purified class II antigens with up to 48 antigens per bead). The beads represented approximately 80 class I antigens and 25 class II antigens and were run simultaneously. Briefly, 5 μL microbeads were incubated with 20 μL sample in a 96-well V-bottomed polystyrene plate (Cytiva, Marlborough, MA, USA) for 30 min in the dark at 20–25 °C with gentle agitation and then washed three times with wash buffer provided by One Lambda. Plates were centrifuged at 1300× *g* for 5 min between each wash step. PE-conjugated anti-human IgG (80 μL) was added for a second 30-min incubation at 20–25 °C in the dark with gentle agitation, followed by two more washes. Negative control serum provided by One Lambda was included in each batch of specimens. Samples were acquired on a luminometer (Luminex, Austin, TX, USA), with the ability to discriminate up to 100 unique beads in one reaction.

#### 4.5.6. Hemagglutination Assays

Anti-A, anti-B, and anti-D hemagglutination (HA) assays were performed according to compendial methods (European Pharmacopoeia) by Haematologic Technologies (Essex Junction, VT, USA) on the GMP lot only. Briefly, red blood cells were washed and then pre-treated with papain. Washed, pretreated A, B, and O type red blood cells were exposed to a serial dilution of the test material alongside positive and negative controls, and each cell type was macroscopically assessed for agglutination at each concentration individually.

#### 4.5.7. Human Tissue Microarrays

A human tissue microarray study was performed under GLP guidelines at WuXi AppTec (Suzhou, China). Tissues (n = 37) were assessed (Table S6).

Commercial EZ-Link Sulfo-NHS-LC-Biotinylation Kit was used for the biotinylation of GIGA-2050 (Tox lot) and IVIG (Gamunex; Grifols, S.A., Sant Cugat del Vallès, Spain). Biotinylated GIGA-2050 and IVIG were used for binding to frozen normal tissues at the concentrations of 2.5 µg/mL and 25 µg/mL using immunohistochemistry staining. SARS-CoV-2 RBD protein provided by WuXi AppTec was used as a positive control target on the arrays. PBS (0.01 mol/L) was used as the negative control reagent. Slides stained with biotinylated GIGA-2050 or IVIG were evaluated to identify the stained tissue elements or cell types and their staining intensity and frequency. Staining patterns of all the cell types were recorded, including cell membrane, cytoplasm, and/or nucleus.

The evaluation criterion for the intensity of staining was as follows: negative, no staining; 1+, weak staining; 2+, moderate staining; 3+, strong staining; 4+, intense staining. Grading of staining frequency were described as follows: very rare, <1% of cells of a particular cell type; rare, 1–5% of cells of a particular cell type; rare to occasional, 5–25% of cells of a particular cell type; occasional, 25–50% of cells of a particular cell type; occasional to frequent, 50–75% of cells of a particular cell type; frequent, 75–100% of cells of a particular cell type. There was no staining in the slides stained with the PBS negative control reagent, GIGA-2050 stained RBD protein on all slides, and IVIG did not stain RBD protein on any slide.

### 4.6. In Vivo Characterization

#### 4.6.1. Nonhuman Primate Toxicokinetics

Nonhuman primate toxicokinetics were performed under GLP guidelines at WuXi AppTec (Suzhou, China) under a protocol reviewed and approved by the local Institutional Animal Care and Use Committee (IACUC). Twenty-four (12/sex) cynomolgus macaques were randomly assigned to four groups of three/sex/group, and they were administered GIGA-2050 once via a 2-h intravenous (IV) infusion at the dosage of 0 (0.9% saline solution), 25, 125, or 475 mg/kg. The dose volume was 1.7 mL/kg for Group 2, 8.5 mL/kg for Group 3, and 32.3 mL/kg for Groups 1 and 4, respectively. At initiation of dosing, male and female NHPs were approximately 2.5 to 3.5 years of age and body weights ranged from 1.9 to 2.3 kg for males and 2.0 to 2.3 kg for females. NHPs were necropsied on Day 29 after dosing.

NHPs were observed for mortality, clinical signs, injection site observations, body weights, food consumption, ophthalmic examinations, body temperatures, safety pharmacology (electrocardiography, blood pressure, heart rate, respiration, and neurological examinations), clinical pathology (hematology, coagulation, serum chemistry, and urinalysis), cytokine analysis, organ weights, and macroscopic and microscopic examinations of gross lesions. Blood was also collected for PK and pharmacodynamics (PD) analysis. Table S8 summarizes the parameters assessed in the TK study.

To assess the TK of GIGA-2050, a validated GLP method for anti-SARS-CoV-2 ELISA (Marin Biologic Laboratories, Novato, CA, USA) was used to determine the antibody concentrations against the SARS-CoV-2 Spike protein in cynomolgus macaque plasma. SARS-CoV-2 Spike protein (Sino Biological, Wayne, PA, USA) was used to coat ELISA plates at 2 μg/mL. Serial dilutions of reference GIGA-2050, QC controls consisting of high, mid, and low concentrations of GIGA-2050, and cynomolgus macaque plasma samples from the TK study were added to the plate. HRP-conjugated goat anti-human IgG antibody cross adsorbed for monkey IgG (Southern Biotech, Birmingham, AL, USA) was used as the detection antibody. Quantitative measurements of antibody binding to SARS-CoV-2 Spike were performed on a microplate reader (Cytation 5; BioTek, Winooski, VT, USA) and antibody levels were interpolated from a four-parameter logistic fit of the standard curve. Anti-SARS-CoV-2 ELISA serum titer has been shown to correlate with neutralizing titer for GIGA-2050 [12].

As a second method to assess the TK of GIGA-2050, a validated GLP method for anti-human IgG ELISA (Marin Biologic Laboratories, Novato, CA, USA) was developed to detect the concentrations of human IgG antibodies in cynomolgus macaque plasma. Goat anti-human IgG antibody cross adsorbed for monkey IgG was used to coat ELISA plates (Southern Biotech, Birmingham, AL, USA). Serial dilutions of reference GIGA-2050, QC controls consisting of high, mid, and low concentrations of GIGA-2050, and cynomolgus macaque plasma samples from the TK study were added to the plate. HRP-conjugated goat anti-human IgG antibody cross adsorbed for monkey IgG (Southern Biotech, Birmingham, AL, USA) was used as detection antibody. Quantitative measurements of antibody binding were performed on a microplate reader (Cytation 5; BioTek, Winooski, VT, USA) and antibody levels were interpolated from a four-parameter logistic fit of the standard curve.

PK analysis of human IgG1 and anti-SARS-CoV-2 serum concentration-time data was performed by non-compartmental approaches using the software package PK (version 1.3–5) in R (Table S7) PK parameter values, including the maximum measured serum concentrations (Cmax), the time to reach the maximum measured concentrations (Tmax), half-life (T1/2), distribution volume at steady state (VSS), and the area under the serum concentration vs. time curve (AUC0-last, AUC0-inf), were determined. The AUC0-last was calculated using the linear trapezoidal rule on the arithmetic means at the different time points while the extrapolation necessary for the AUC0-inf, and AUC0-inf was calculated assuming an exponential decay on the last time points. Male and female PK data were analyzed separately.

#### 4.6.2. Mouse In Vivo Efficacy

GIGA-2050 in vivo efficacy was performed by the Coronavirus Immunotherapy Consortium (CoVIC; https://covicdb.lji.org accessed on 5 May 2022), an international effort to conduct side-by-side analyses of candidate antibody therapeutics targeting the SARS-CoV-2 spike protein in standardized assays. This mouse study was performed under a protocol reviewed and approved by the Texas Biomedical Research Institute IACUC protocol number #1745 MU. The in vivo model was established to demonstrate protective efficacy with highly neutralizing antibodies. The K18 hACE2 transgenic mouse (#034860; The Jackson Labs, Bar Harbor, ME, USA) model was used; this highly pathogenic model was lethal by Day 5 after infection with SARS-CoV-2 [15]. One day before challenging with virus (Day −1), ten K18 hACE2 transgenic mice received 1.5 mg/kg of the monoclonal antibody CC12.3, known to have neutralizing efficacy, and this was used as a reference control [22]. One day before challenging with virus (Day −1), ten K18 hACE2 transgenic mice received 5 mg/kg of GIGA-2050. Both drugs were administered intraperitoneally (IP). Five infected but untreated mice and five untreated and uninfected mice were included as study controls. A lethal dose of 1 × 10^5^ PFU of SARS-CoV-2/human/WA-CDC-WA1/2020 passage 6 (MC985325) was delivered intranasally (IN) in 50 μL volume per animal (Day 0) and the animals were monitored for morbidity (weight loss) and mortality (survival) [15]. Following our approved IACUC protocol, mice presenting >25% body weight loss were humanely euthanized. Log-rank (Mantel-Cox) test for pairwise comparison of Kaplan–Meier survival curves and log-rank Hazard Ratios were performed with Prism (GraphPad, San Diego, CA, USA). On Day 0, 24 h after drug administration and immediately before virus challenge, serum samples were collected and an ELISA targeting the pre-spike SARS-CoV-2 antigen was performed (Nexelis, Laval, QC, Canada). The serum concentration of GIGA-2050 was 1756.3 binding antibody units (BAU)/mL ± 241.9, and the serum concentration of mAb CC12.3 was 752.0 BAU/mL ± 82.8 (mean ± standard error of the mean).

## Figures and Tables

**Table 1:** Release data for development, toxicology, and GMP lots of GIGA-2050. HMWS, high molecular weight species. LMWS, low molecular weight species. ND, Not determined.

		Acceptance Criteria	Dev-A	Dev-B	Dev-C	Dev-D	Dev-E	Dev-F	Tox	GMP
Purity	Native size distribution by SEC-HPLC	Monomer: ≥90% Main Peak	98.3%	97.9%	98.7%	99.4%	96.7%	98%	99.6%	98%
		HMWS: Report	1.3%	1.6%	1.3%	0.5%	3.2%	1.6%	0.4%	1.4%
		LMWS: Report	0.4%	0.5%	0%	0.1%	0.1%	0.0%	0%	0.7%
	Denatured size distribution by CE-SDS (non-reduced)	>85% Intact	92.5%	93.9%	91%	87.3%	93.5%	95%	85.2%	94%
	Denatured size distribution by CE-SDS (reduced)	>85% Heavy Chain + Light Chain	100%	99.8%	100%	99.8%	100%	98%	100%	98%
	Residual CHO DNA	<1 pg/mg	ND	ND	<1.1 pg/mg	ND	ND	<0.6 pg/mg	<0.9 pg/mg	<0.7 pg/mg
	Residual CHO HCP ELISA	<50 ppm	5.6 ppm	6.3 ppm	11.5 ppm	3.8 ppm	11.4 ppm	<1.9 ppm	3.0 ppm	8.0 ppm
Potency	Anti-SARS-CoV-2 binding ELISA	Binding: EC50 = 0.03–0.05 mg/L	0.028–0.031	0.035–0.039	0.043–0.046	0.036–0.039	0.034–0.036	0.049–0.054	0.041–0.043	0.047–0.050
	SARS-CoV-2 pseudotype neutralization potency assay	Potency: IC50 = 0.3–0.5 mg/L	0.31–0.37	0.37–0.49	0.34–0.38	0.36–0.43	0.28–0.35	0.42–0.53	0.31–0.38	0.42–0.48

## Data Availability

Sequencing data are available in the Short Read Archive (SRA) under project identifier PRJNA784610.

## Supplementary Materials

The following supporting information can be downloaded at: https://www.mdpi.com/article/10.3390/pathogens11070806/s1, Figure S1: Viable cell density, viability, and antibody production of the mock MCB from P0 to P20, Figure S2: SEC data from the aged mock MCB, Figure S3: CE-SDS data from the aged mock MCB, Figure S4: SARS-CoV-2 ELISA data from the aged mock MCB, Figure S5: Concentration of GIGA-2050 in cynomolgus plasma samples, Figure S6: Body weight change from the mouse in vivo efficacy study, Table S1: Vessel sizes and types used for fed-batch production of GIGA-2050 development, toxicology, and GMP lots, Table S2: Copy number, product quality, and potency data for the mock MCB stability study, Table S3: Product quality and impurity data for concentrated clarified harvest samples from the Dev-F and GMP lots, Table S4: Product quality and impurity data for viral inactivation pool samples from the Dev-F and GMP lots, Table S5: Product quality and impurity data for cation exchange eluate pool samples from the Dev-F and GMP lots, Table S6: Tissues assessed in the human tissue microarray study, Table S7: PK parameters measured in the cynomolgus TK study, Table S8: Parameters assessed in the cynomolgus TK study.
