# Supplementary material for: GMP Manufacturing and IND-Enabling Studies of a Recombinant Hyperimmune Globulin Targeting SARS-CoV-2
Source: Pathogens. 2022 Jul 19;11(7):806. doi: 10.3390/pathogens11070806 (PMC9320065; doi:10.3390/pathogens11070806)
Supplement: Supplementary file 1 [file pathogens-11-00806-s001.zip › Supplementary Figures-revised.pptx]

## Slide 1
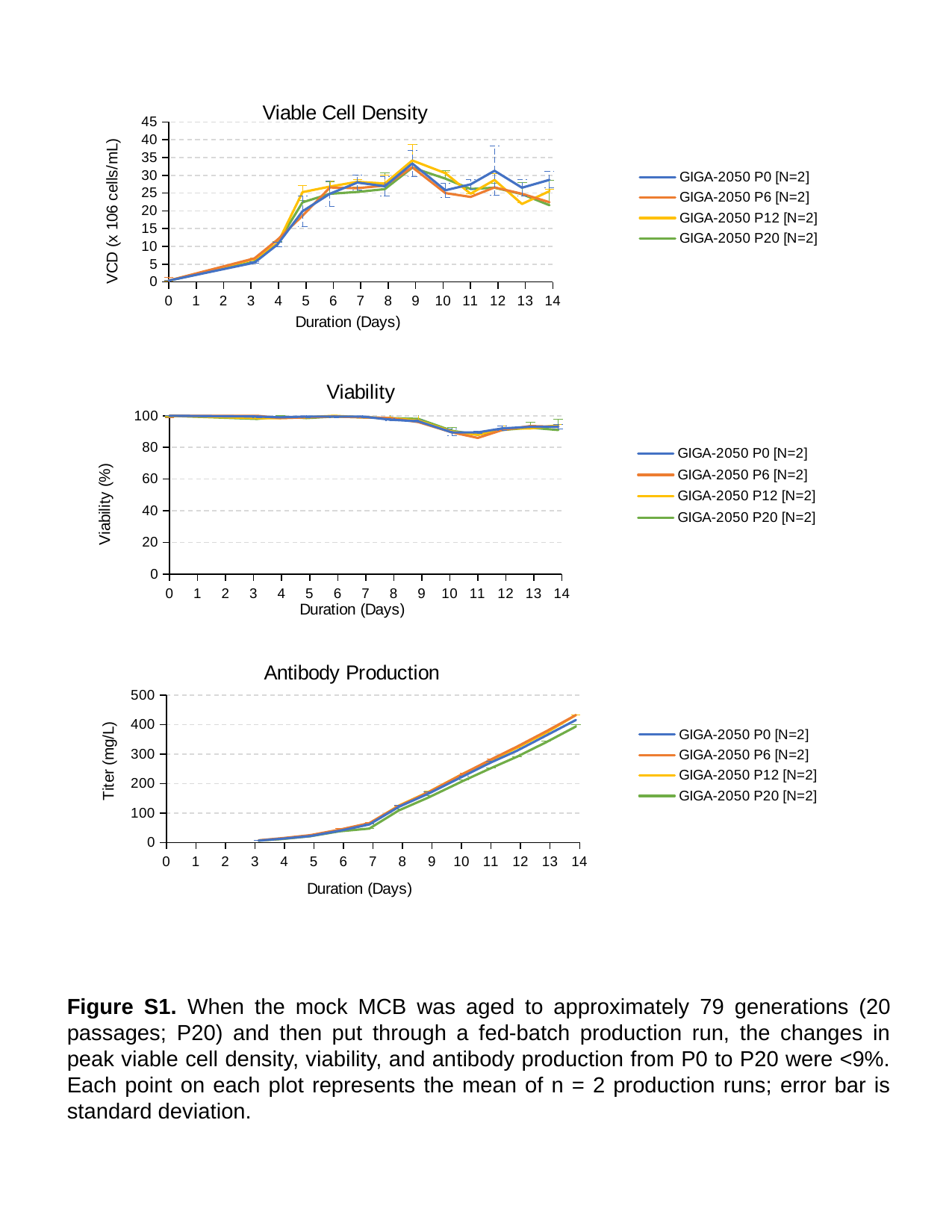

### Chart: Viable Cell Density
| Category | GIGA-2050 P0 [N=2] | GIGA-2050 P6 [N=2] | GIGA-2050 P12 [N=2] | GIGA-2050 P20 [N=2] |
|---|---|---|---|---|
### Chart: Viability
| Category | GIGA-2050 P0 [N=2] | GIGA-2050 P6 [N=2] | GIGA-2050 P12 [N=2] | GIGA-2050 P20 [N=2] |
|---|---|---|---|---|
### Chart: Antibody Production
| Category | GIGA-2050 P0 [N=2] | GIGA-2050 P6 [N=2] | GIGA-2050 P12 [N=2] | GIGA-2050 P20 [N=2] |
|---|---|---|---|---|Figure S1. When the mock MCB was aged to approximately 79 generations (20 passages; P20) and then put through a fed-batch production run, the changes in peak viable cell density, viability, and antibody production from P0 to P20 were <9%. Each point on each plot represents the mean of n = 2 production runs; error bar is standard deviation.

## Slide 2
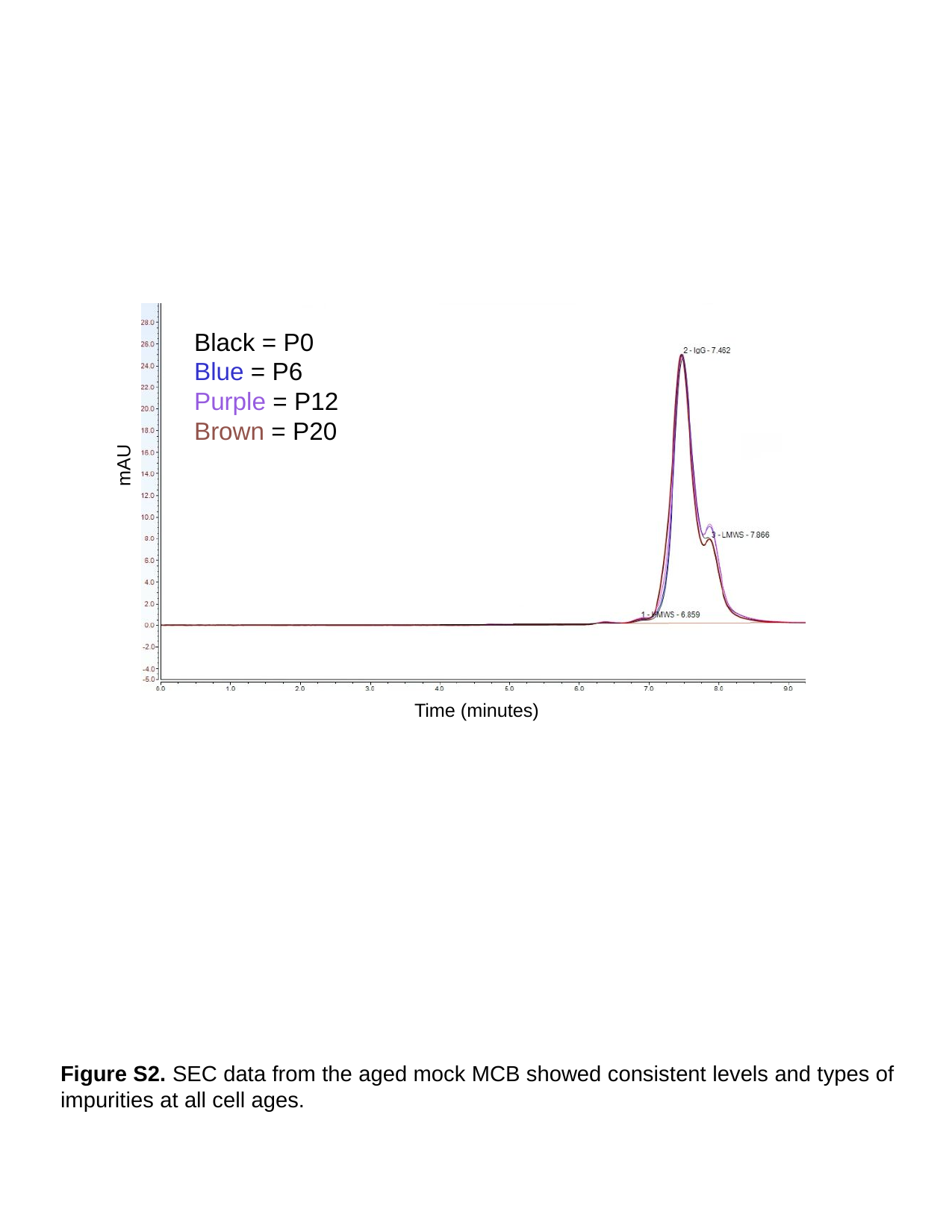

Black = P0
Blue = P6
Purple = P12
Brown = P20
mAU
Time (minutes)
Figure S2. SEC data from the aged mock MCB showed consistent levels and types of impurities at all cell ages.

## Slide 3
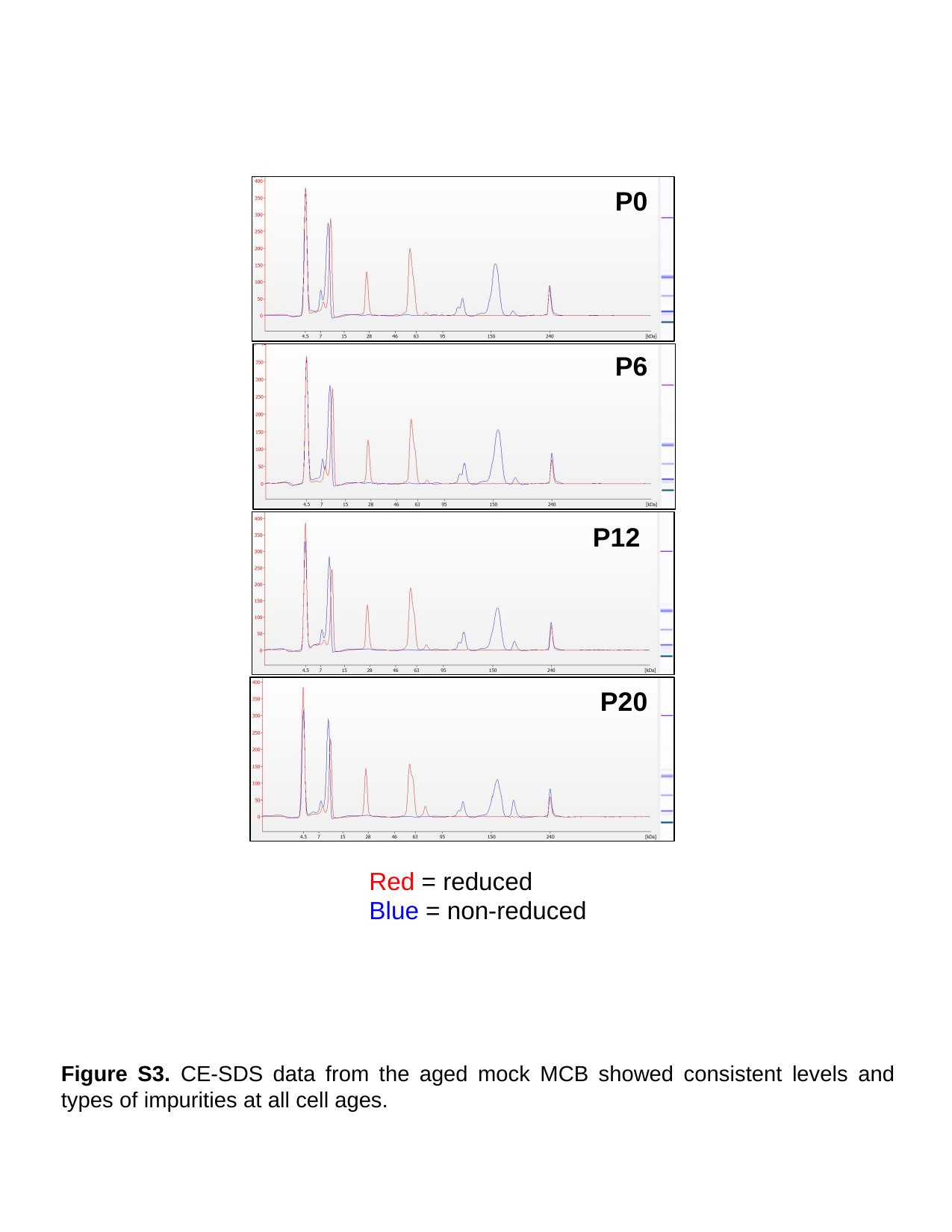

P0
P6
P12
P20
Red = reduced
Blue = non-reduced
Figure S3. CE-SDS data from the aged mock MCB showed consistent levels and types of impurities at all cell ages.

## Slide 4
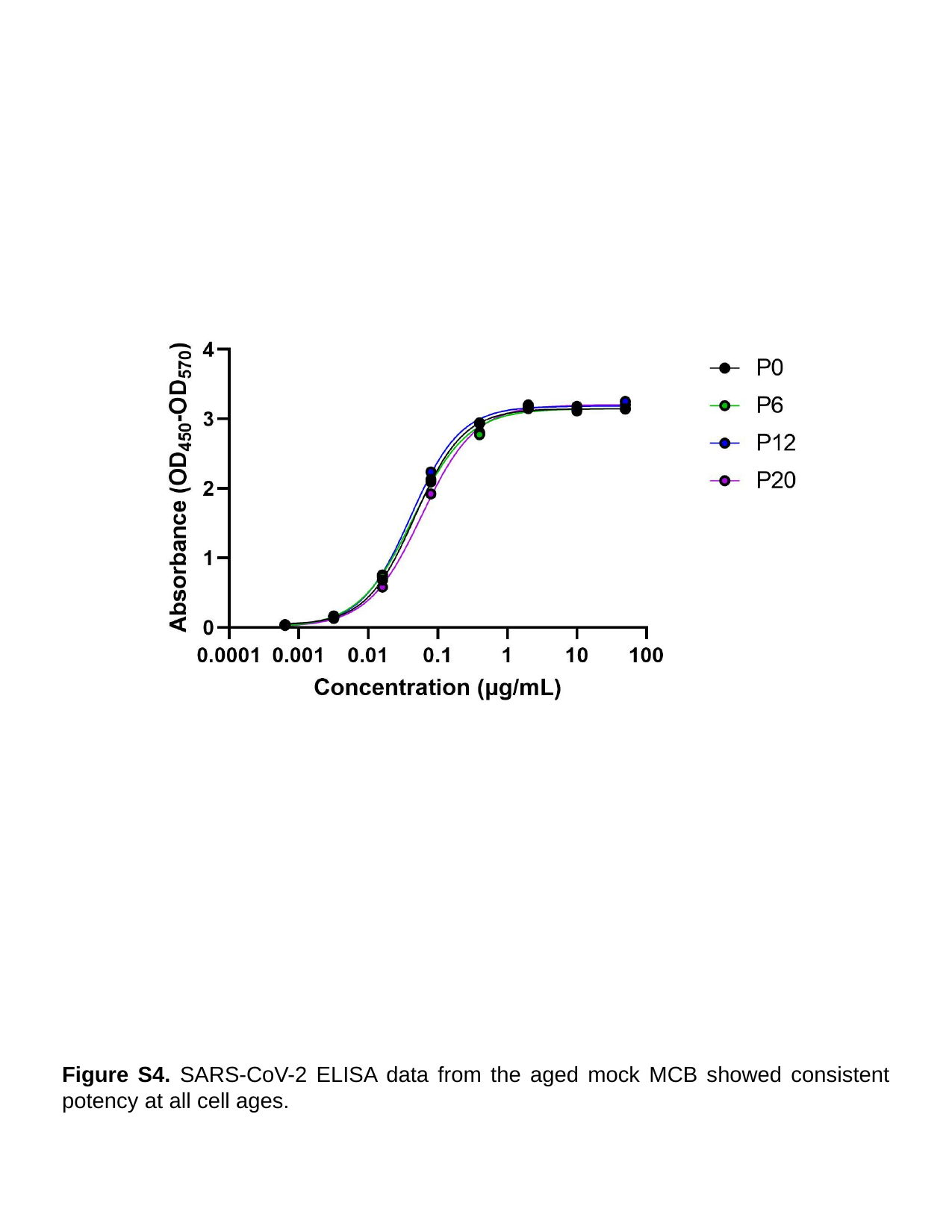

Figure S4. SARS-CoV-2 ELISA data from the aged mock MCB showed consistent potency at all cell ages.

## Slide 5
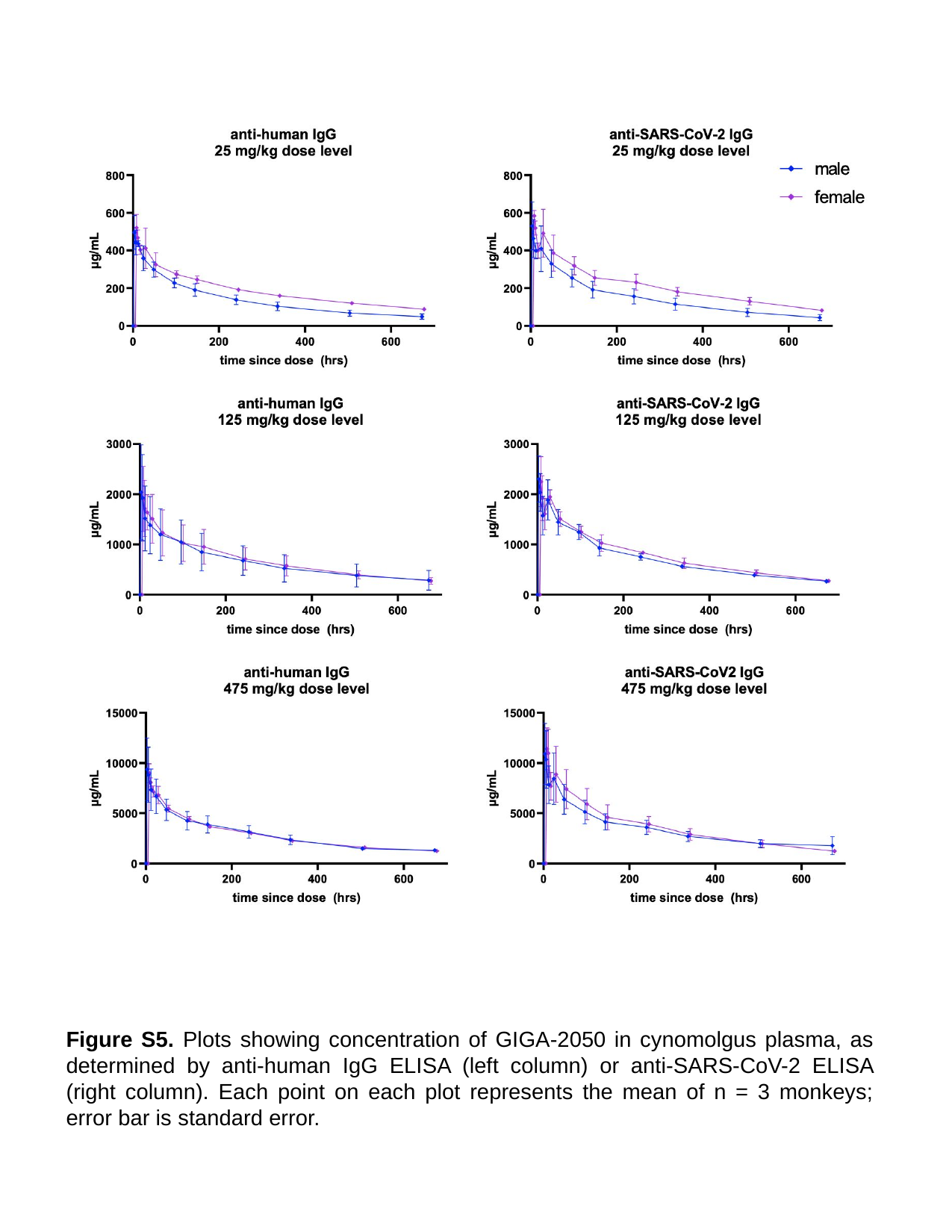

Figure S5. Plots showing concentration of GIGA-2050 in cynomolgus plasma, as determined by anti-human IgG ELISA (left column) or anti-SARS-CoV-2 ELISA (right column). Each point on each plot represents the mean of n = 3 monkeys; error bar is standard error.

## Slide 6
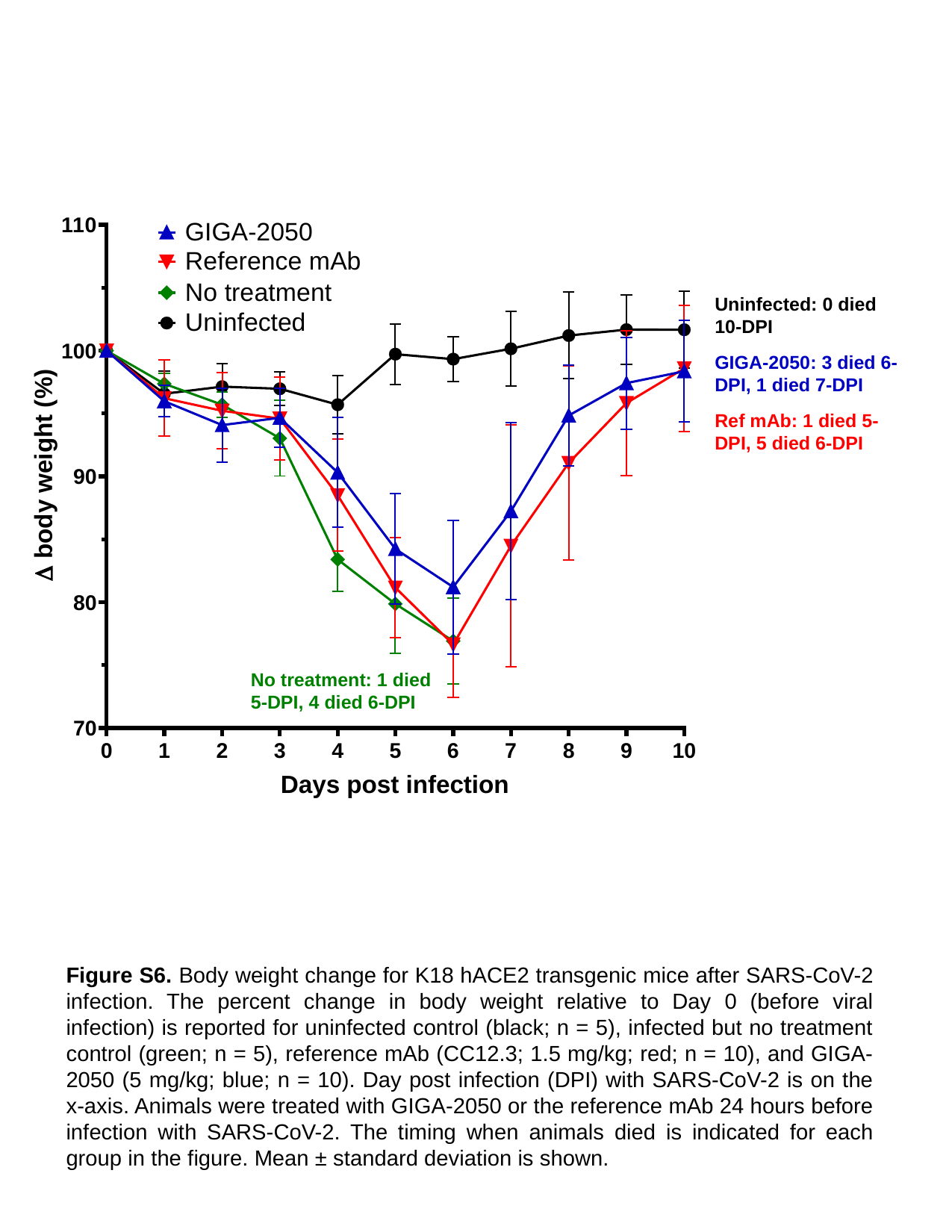

Uninfected: 0 died 10-DPI
GIGA-2050: 3 died 6-DPI, 1 died 7-DPI
Ref mAb: 1 died 5-DPI, 5 died 6-DPI
D body weight (%)
No treatment: 1 died 5-DPI, 4 died 6-DPI
Days post infection
Figure S6. Body weight change for K18 hACE2 transgenic mice after SARS-CoV-2 infection. The percent change in body weight relative to Day 0 (before viral infection) is reported for uninfected control (black; n = 5), infected but no treatment control (green; n = 5), reference mAb (CC12.3; 1.5 mg/kg; red; n = 10), and GIGA-2050 (5 mg/kg; blue; n = 10). Day post infection (DPI) with SARS-CoV-2 is on the x-axis. Animals were treated with GIGA-2050 or the reference mAb 24 hours before infection with SARS-CoV-2. The timing when animals died is indicated for each group in the figure. Mean ± standard deviation is shown.
